# Mental Health First Aid Training Amongst UK Medical Students: A Mixed-Methods Study

**DOI:** 10.1192/bjo.2026.11180

**Published:** 2026-06-30

**Authors:** Su Ying Yeoh, Verity Williams, Jacqueline Robbins, Helen Bintley, Joanne Rodda

**Affiliations:** 1 Kent and Medway Mental Health NHS Trust, Canterbury, United Kingdom; 2 Kent and Medway Medical School, Canterbury, United Kingdom

## Abstract

**Aims:**

Medical students experience higher rates of mental health problems compared with the wider student population. Stigma and fitness-to-practise concerns can deter help-seeking, with students often turning to peers. Mental Health First Aid (MHFA) is a psycho-educational programme developed for the general public, designed to support recognition of mental health difficulties and responses to people in distress. We present an innovative, co-constructed mixed-methods study exploring medical students’ experiences of MHFA training,delivered as a student-selected component within a medical school curriculum. We aimed to explore the motivators, enablers and barriers to student engagement, and students’ perceptions of the value and relevance of MHFA in relation to mental health knowledge, self-care, help-seeking and supporting others. The study also sought to inform the development of a bespoke course tailored to the needs of medical students.

**Methods:**

MHFA was delivered in four half-day face-to-face sessions to Year 1 and 2 medical students at Kent and Medway Medical School. Data were collected via post-session questionnaires, in addition to post-course student-led focus groups and optional semi-structured individual interviews. Questionnaire data were analysed using descriptive statistics, and qualitative data were analysed thematically.

**Results:**

Twenty-five students participated; 21 attended all four sessions (range was 21–25 students). In post-session questionnaires, students rated MHFA content positively in relation to self-care and supporting others, and noted areas of overlap with the medical curriculum, including psychosis, suicide and communication skills. Analysis of qualitative data from four focus groups (n=22) and nine individual interviews identified common themes. Students described generally positive experiences of MHFA training and valued having an adaptable framework for guiding conversations about mental health in professional and personal contexts. An additional theme related to suggestions for adapting the course to better suit the needs of medical students. These included a faster-paced format, case scenarios that more closely reflected medical student experiences, greater integration of skills practice, and increased focus on issues specific to medical students, such as disclosure of mental illness and fitness to practise, exam-related anxiety, and inclusion of doctors’ lived experience of mental illness.

**Conclusion:**

MHFA training was generally experienced positively by medical students and perceived as relevant to self-care and supporting others. Students also identified limitations in how well the course aligned with the specific contexts and pressures of medical training. Together, these findings support the development of a bespoke mental health support course for medical students, building on MHFA approaches while addressing discipline-specific needs.

